# Supplementary material for: Broad receptor tropism and immunogenicity of a clade 3 sarbecovirus
Source: bioRxiv. 2023 Sep 13:2023.09.12.557371. Preprint. [Version 1] doi: 10.1101/2023.09.12.557371 (PMC10515872; doi:10.1101/2023.09.12.557371)
Supplement: Supplement 1 [file NIHPP2023.09.12.557371v1-supplement-1.pdf]

bioRxiv preprint doi: https://doi.org/10.1101/2023.09.12.557371; this version posted September 13, 2023. The copyright holder for this preprint (which was not certified by peer review) is the author/funder, who has granted bioRxiv a license to display the preprint in perpetuity. It is made available under aCC-BY 4.0 International license.

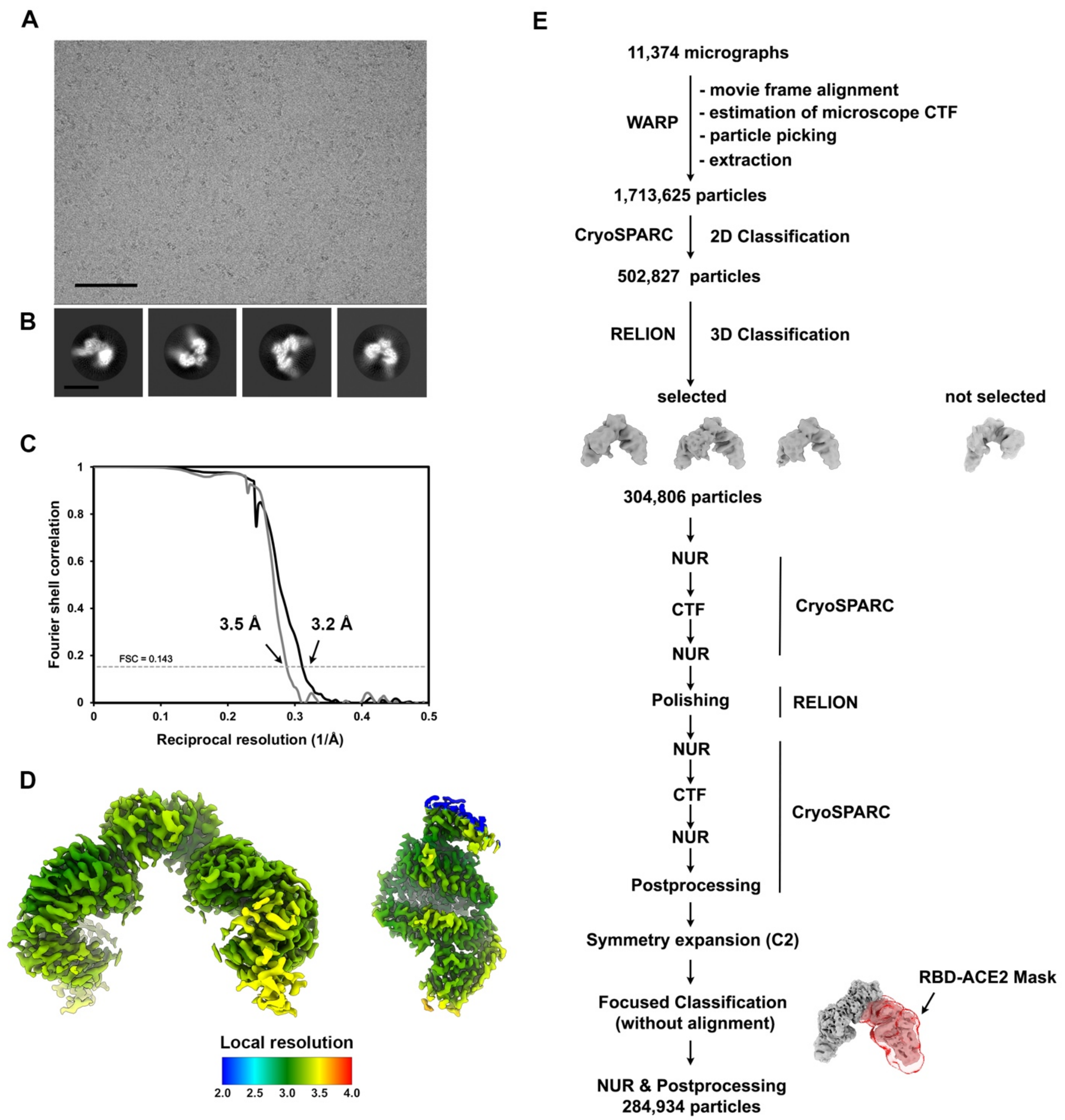

Figure S1 CryoEM data collection and refinement of the dimeric *R. alcyone* ACE2-bound PRD-0038 RBD complex.

(A and B) Representative electron micrograph (A) and 2D class averages (B) of -the dimeric *R. alcyone* ACE2-bound PRD-0038 RBD embedded in vitreous ice. The scale bar represents 100 nm (A) or 100 Å (B).

(C) Gold-standard Fourier shell correlation curves for the final cryoEM reconstructions of the dimeric -ACE2/RBD complex (solid gray line) and locally refined ACE2/RBD (solid black line) shown in (D). The 0.143 cutoff is indicated with a gray dashed line.

(D) Local resolution map calculated using CryoSPARC and plotted onto the sharpened cryoEM map.

(E) Data processing flowchart. CTF: contrast transfer function; NUR: non-uniform refinement.

bioRxiv preprint doi: https://doi.org/10.1101/2023.09.12.557371; this version posted September 13, 2023. The copyright holder for this preprint (which was not certified by peer review) is the author/funder, who has granted bioRxiv a license to display the preprint in perpetuity. It is made available under aCC-BY 4.0 International license.

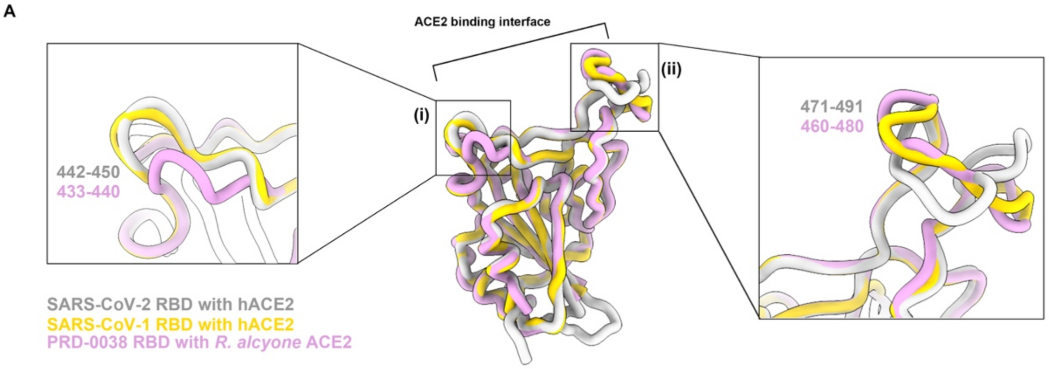

B

(i)

	442	443	444	445	446	447	448	449	450
SARS-CoV-2	D	S	K	V	G	G	N	Y	N
SARS-CoV-1	D	A	T	S	T	G	N	Y	N
PRD-0038	D	S	K	Q	G	N	N	-	-
	433	434	435	436	437	438	439		

(ii)

	471	472	473	474	475	476	477	478	479	480	481	482	483	484	485	486	487	488	489	490	491
SARS-CoV-2	E	I	Y	Q	A	G	S	T	P	C	N	G	V	E	G	F	N	C	Y	F	P
SARS-CoV-1	V	P	F	S	P	D	G	K	P	C	T	P	-	P	A	L	N	C	Y	W	P
PRD-0038	V	L	Y	N	S	A	G	G	T	C	S	S	T	S	Q	L	G	C	Y	E	P
	460	461	462	463	464	465	466	467	468	469	470	471	472	473	474	475	476	477	478	479	480

Figure S2 Structural distinctions between the PRD-0038, SARS-CoV-2 and SARS-CoV-1 RBDs near the ACE2-binding interface (RBM).

(A) Ribbon diagrams showing a superimposition of the *R. alcyone* ACE2-bound PRD-0038 RBD (pink) superimposed to the human ACE2-bound SARS-CoV-2 RBD (gray, PDB 6M0J[33]) and SARS-CoV-1 RBD (gold, PDB 2AJF[34]) structures (ACE2s not shown for clarity). Insets: close-up views of two RBM regions.

(B) Amino acid sequence alignment of the SARS-CoV-2, SARS-CoV-1, and PRD-0038 RBD regions highlighted in the insets shown in panel (A). - indicate deletions.

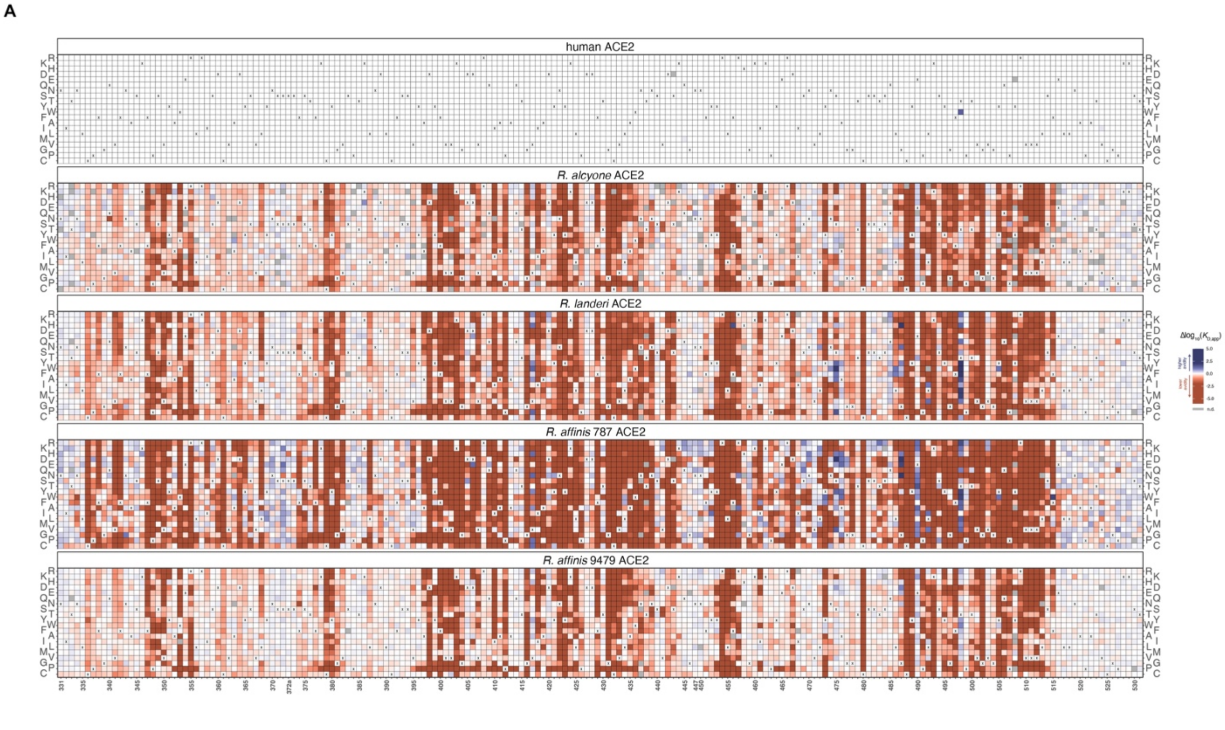

Figure S3 Heatmaps of change in ACE2-binding avidity resulting from RBD mutations determined by DMS.

bioRxiv preprint doi: https://doi.org/10.1101/2023.09.12.557371; this version posted September 13, 2023. The copyright holder for this preprint (which was not certified by peer review) is the author/funder, who has granted bioRxiv a license to display the preprint in perpetuity. It is made available under aCC-BY 4.0 International license.

(A) Change in ACE2-binding avidity for hACE2, *R. alcyone* ACE2, and *R. landeri* ACE2 (delta-$\log_{10}K_{D}$,apparent). Residue numbering corresponds to SARS-CoV-2.

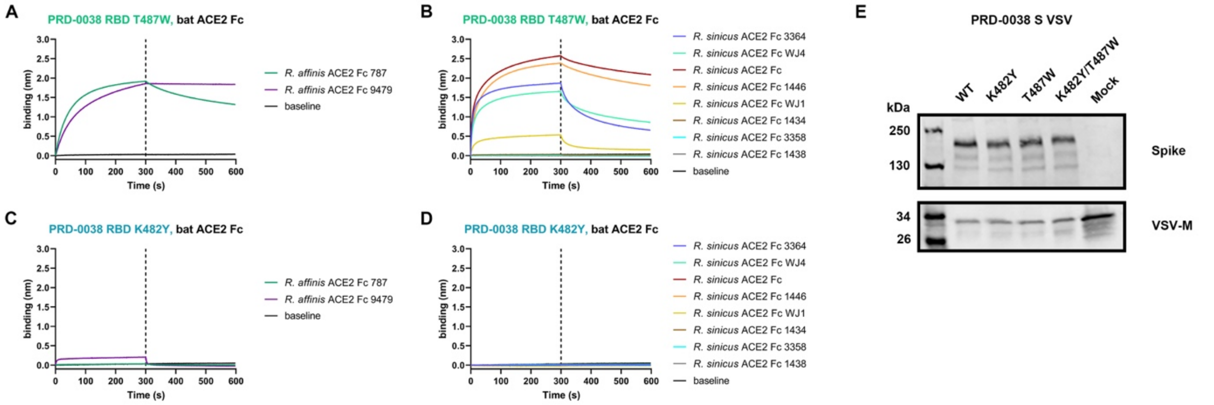

Figure S4 Binding data and Western blot analysis.

(A and B) BLI binding analysis of 1 μM dimeric *R. affinis* (A) and *R. sinicus* (B) ACE2-Fc alleles to the biotinylated T487W PRD-0038 RBD immobilized on streptavidin biosensors.

(C and D) BLI binding analysis of 1 μM dimeric *R. affinis* (C) and *R. sinicus* (D) ACE2-Fc alleles to the biotinylated K482Y PRD-0038 RBD immobilized on streptavidin biosensors.

(E) Representative Western Blot of wildtype (WT) and mutant PRD-0038 S VSV pseudoviruses normalized based on the amount of incorporated S and VSV-M. Anti-VSV-M Antibody (Kerafast) and Monoclonal ANTI-FLAG® M2 antibody produced in mouse (Sigma) were used as the primary antibody against VSV backbone and S, respectively. Alexa Fluor® 680 AffiniPure Goat Anti-Mouse IgG (Jackson ImmunoResearch) was used as the secondary antibody.

bioRxiv preprint doi: https://doi.org/10.1101/2023.09.12.557371; this version posted September 13, 2023. The copyright holder for this preprint (which was not certified by peer review) is the author/funder, who has granted bioRxiv a license to display the preprint in perpetuity. It is made available under aCC-BY 4.0 International license.

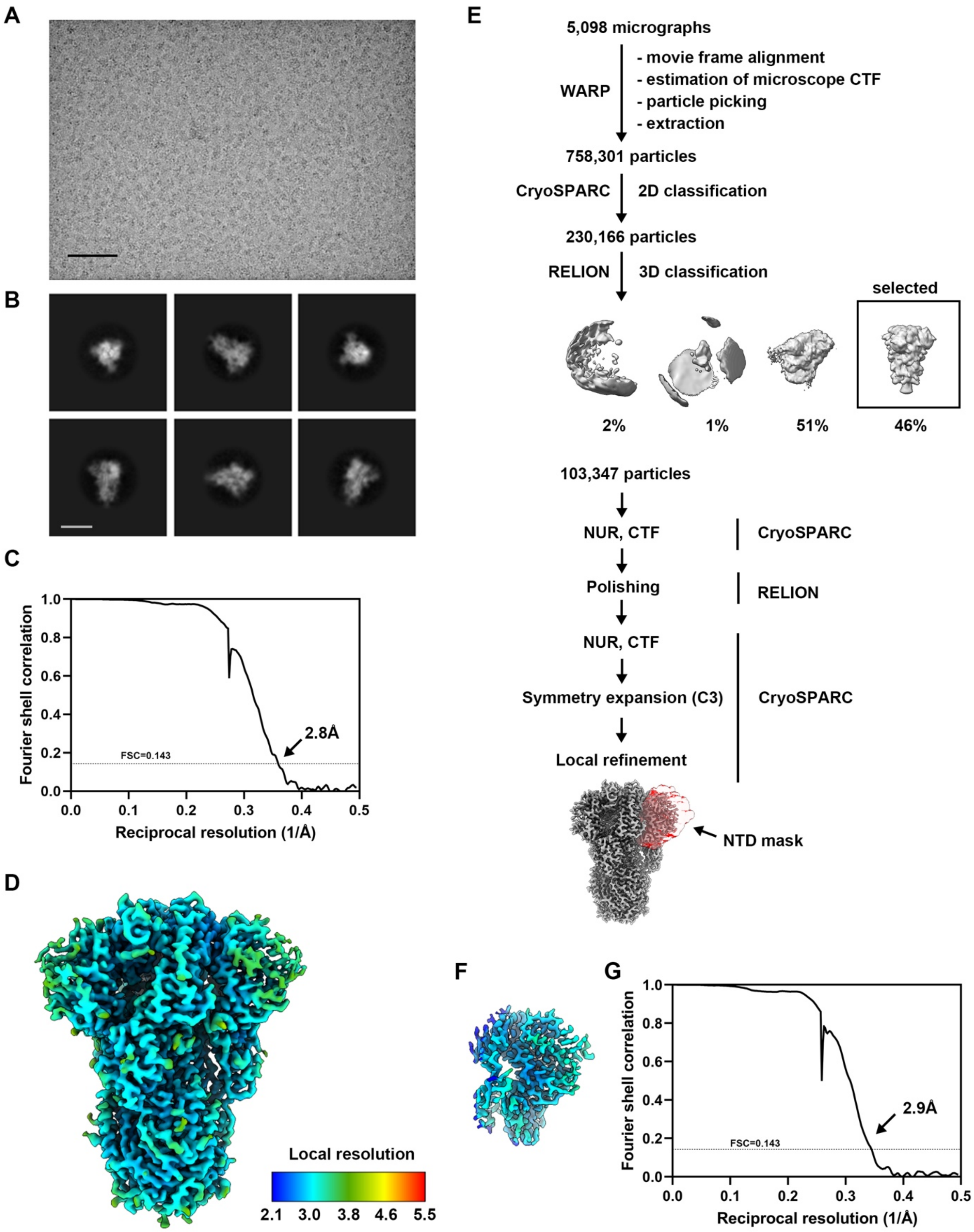

Figure S5 CryoEM data collection and refinement of PRD-0038 S.
(A and B) Representative electron micrograph (A) and 2D class averages (B) of PRD-0038 PentaPro S embedded in vitreous ice. The scale bar represents 100 nm (A) or 100Å (B).

bioRxiv preprint doi: https://doi.org/10.1101/2023.09.12.557371; this version posted September 13, 2023. The copyright holder for this preprint (which was not certified by peer review) is the author/funder, who has granted bioRxiv a license to display the preprint in perpetuity. It is made available under aCC-BY 4.0 International license.

(C) Gold-standard Fourier shell correlation curve for the cryoEM reconstruction. The 0.143 cutoff is indicated with a gray dashed line.

(D) 3D reconstruction of PRD-0038 PentaPro S colored by local resolution as determined using cryoSPARC.

(E) Data processing flowchart. CTF: contrast transfer function; NUR: non-uniform refinement.

(F) 3D reconstruction obtained by local refinement of the PRD-0038 S NTD colored by local resolution as determined using cryoSPARC.

(G) Gold-standard Fourier shell correlation curve. The 0.143 cutoff is indicated with a gray dashed line.

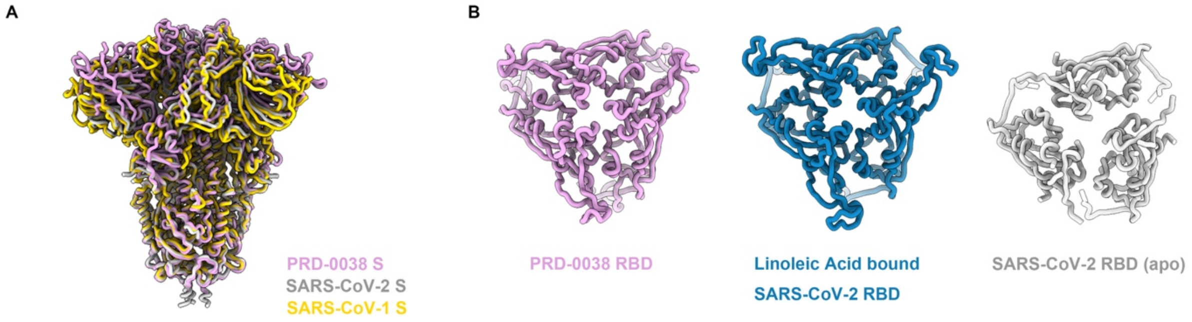

Figure S6 Structural resemblance of PRD-0038 S to SARS-CoV-2 and SARS-CoV-1.

(A) Ribbon diagram of the PRD-0038 S trimer superimposed to SARS-CoV-2 S (PDB 6VXX[5]) and SARS-CoV-1 S (PDB 5X5B[43]).

(B) Contact among RBDs within S trimers viewed from the apex along the 3-fold molecular axis of PRD-0038 S (pink), linoleic acid-bound SARS-CoV-2 S (blue, PDB 6ZB5[44]), and apo SARS-CoV-2 S (gray, PDB 6VXX[5]).

bioRxiv preprint doi: https://doi.org/10.1101/2023.09.12.557371; this version posted September 13, 2023. The copyright holder for this preprint (which was not certified by peer review) is the author/funder, who has granted bioRxiv a license to display the preprint in perpetuity. It is made available under aCC-BY 4.0 International license.

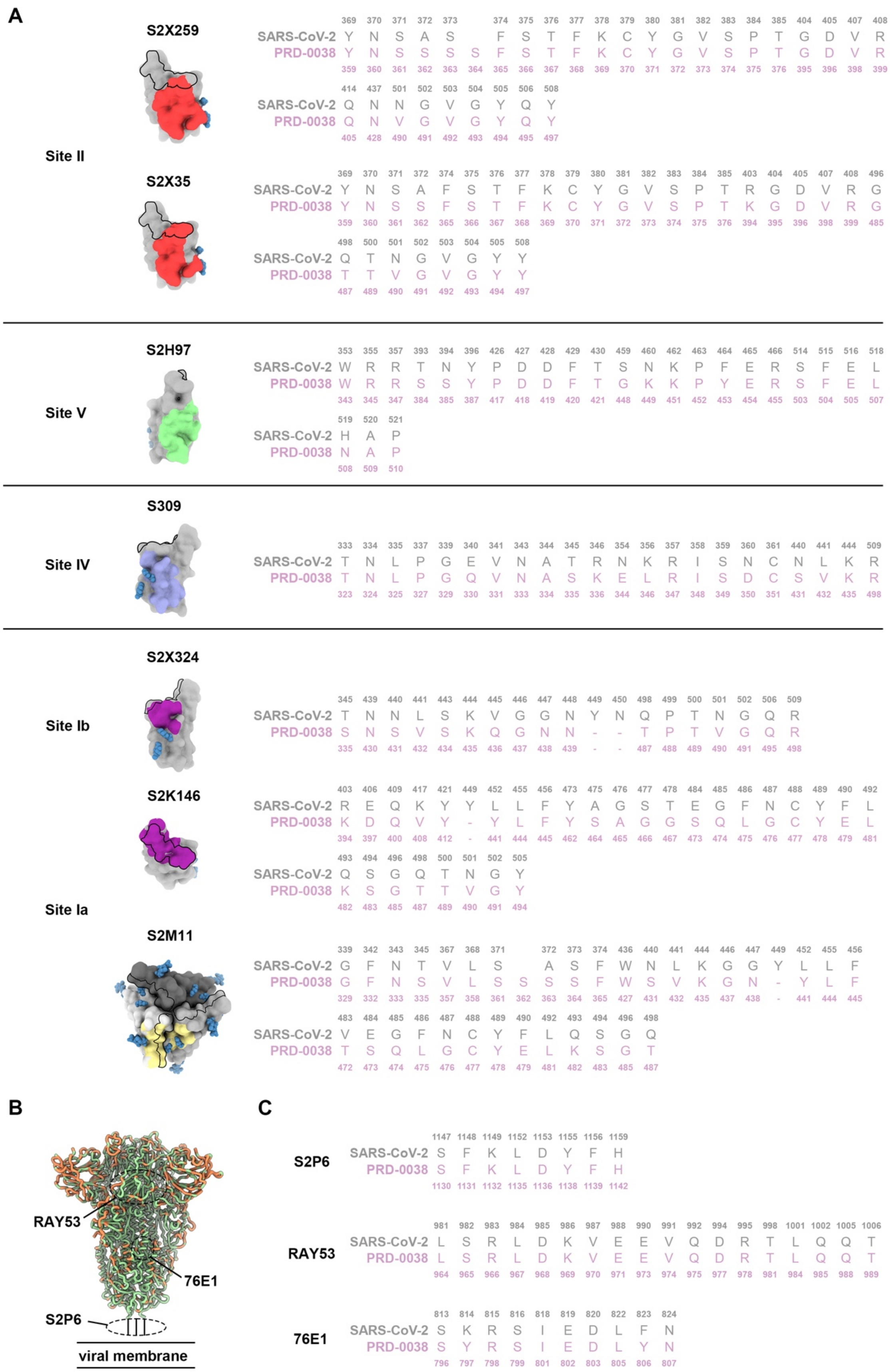

Figure S7

bioRxiv preprint doi: https://doi.org/10.1101/2023.09.12.557371; this version posted September 13, 2023. The copyright holder for this preprint (which was not certified by peer review) is the author/funder, who has granted bioRxiv a license to display the preprint in perpetuity. It is made available under aCC-BY 4.0 International license.

Conservation analysis of epitopes targeted by monoclonal antibodies between PRD-0038 S and SARS-CoV-2 S.

(A) Epitope mapped onto PRD-0038 RBD structure with sequence alignment of key residues at the interface. The PRD-0038 RBD is shown in gray (three RBDs are shown with distinct shades of gray for S2M11 which recognizes a quaternary epitope) and N-linked glycans are rendered as blue spheres. SARS-CoV-2 residue numbering is shown in gray and PRD-0038 residue numbering is shown in pink.

(B) PRD-0038 S sequence conservation with SARS-CoV-2 S at key S_2 fusion machinery epitopes (indicated with dashed lines). Residues are colored according to sequence identity (orange : not conserved, green : conserved).

(C) Sequence alignment of corresponding S_2 epitopes. SARS-CoV-2 residue numbering is shown in gray and PRD-0038 residue numbering is shown in pink. For (A) and (C) the RBM is depicted with a black outline and the monoclonal antibody epitopes are colored according to their antigenic sites (I, purple; II, red; IV, violet; V, green).

bioRxiv preprint doi: https://doi.org/10.1101/2023.09.12.557371; this version posted September 13, 2023. The copyright holder for this preprint (which was not certified by peer review) is the author/funder, who has granted bioRxiv a license to display the preprint in perpetuity. It is made available under aCC-BY 4.0 International license.

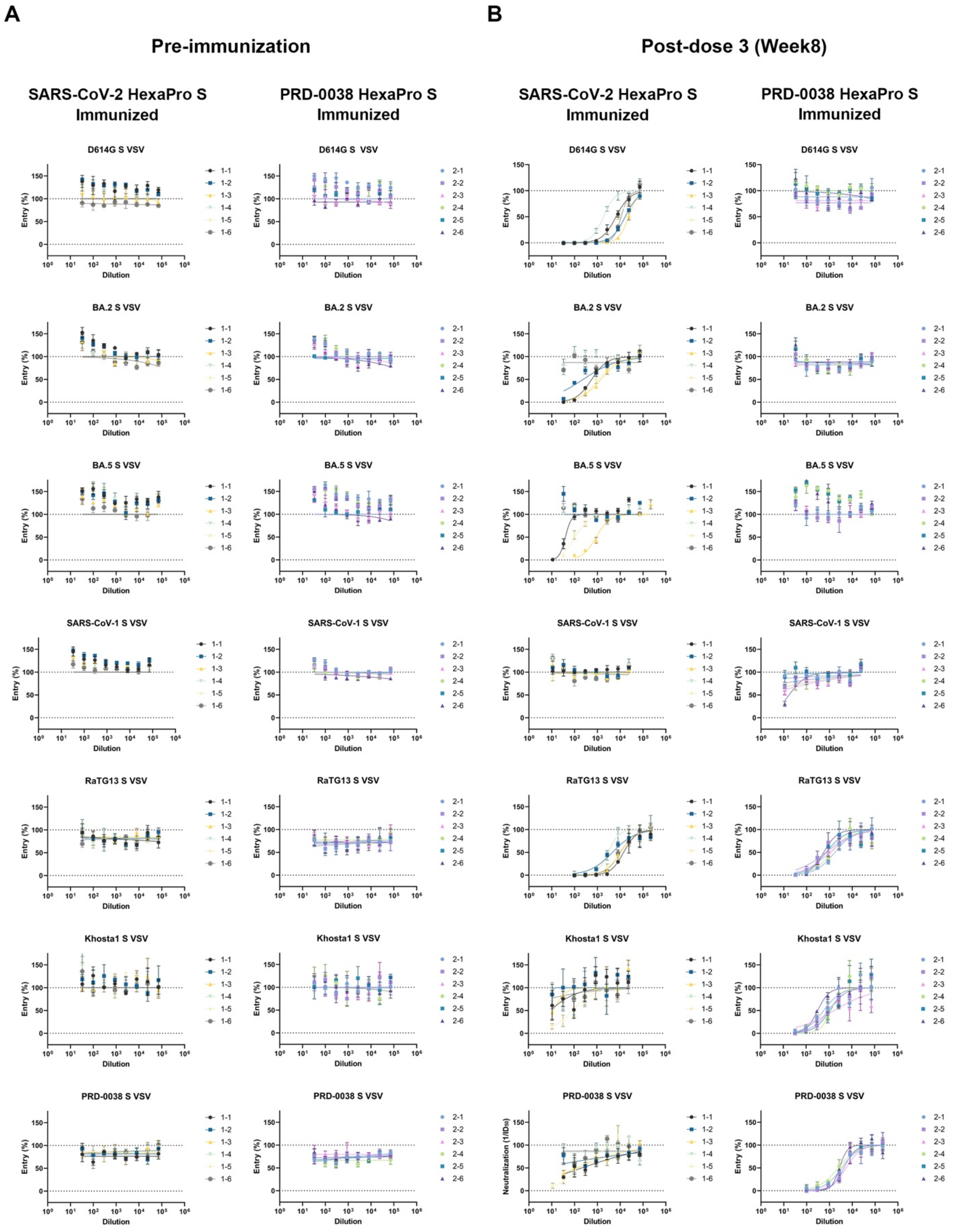

Figure S8 Dose-response curves of mouse serum neutralization before (A) and after (B) three immunizations with SARS-CoV-2 HexaPro S or PRD-0038 HexaPro S.

bioRxiv preprint doi: https://doi.org/10.1101/2023.09.12.557371; this version posted September 13, 2023. The copyright holder for this preprint (which was not certified by peer review) is the author/funder, who has granted bioRxiv a license to display the preprint in perpetuity. It is made available under aCC-BY 4.0 International license.

Neutralization of the indicated sarbecovirus S VSV pseudotypes was assessed for each animal serum two weeks post dose 3 (week 8), as indicated by the color key.

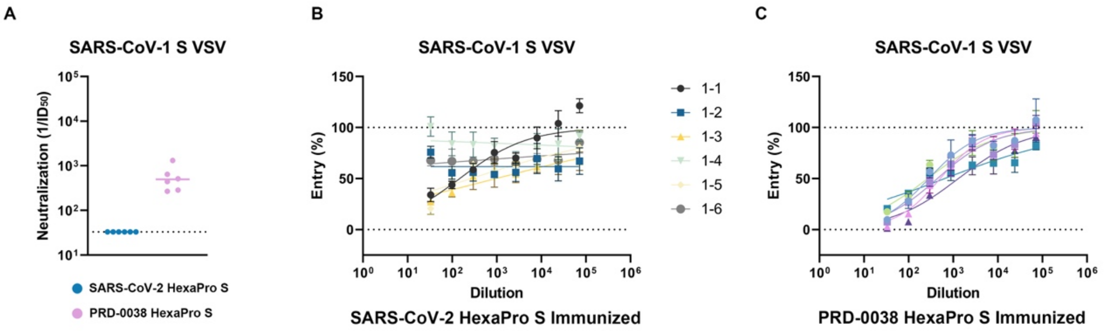

Figure S9 Neutralization of SARS-CoV-1 S VSV by vaccine-elicited mouse sera using a highly diluted pseudovirus input.
(A) Reciprocal ID_{50} value.
(B and C) Dose-response curves for neutralization of SARS-CoV-1 S VSV by SARS-CoV-2 HexaPro S-elicited sera (B) and PRD-0038 HexaPro S-elicited sera (C).

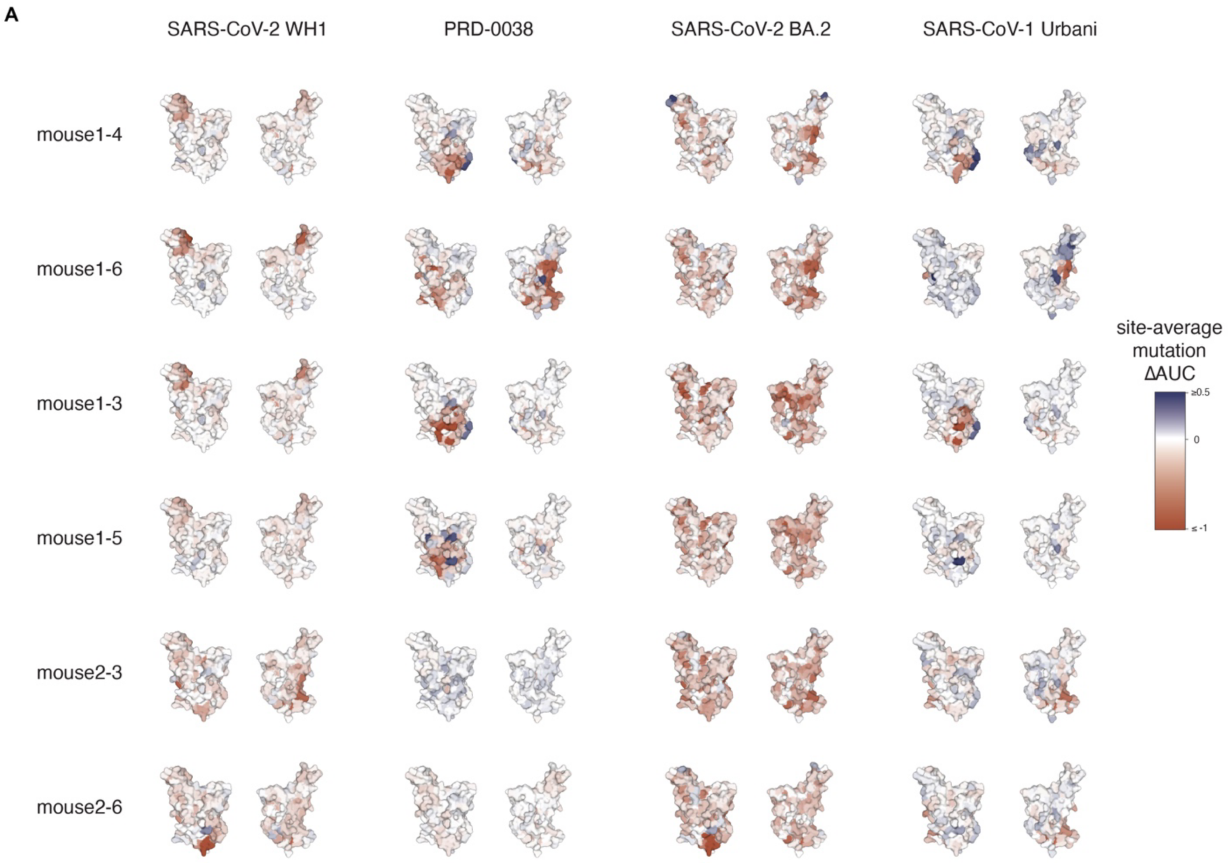

Figure S10 DMS epitope mapping of serum antibodies.
(A) Evaluation of epitopes targeted by serum antibodies elicited by SARS-CoV-2 HexaPro S (mouse 1-3, 1-4, 1-5, and 1-6) or PRD-0038 HexaPro S (mouse 2-3 and 2-6) vaccination using yeast-displayed DMS of vaccine-matched and mismatched RBDs (indicated above each column). The average effect of mutations at each site are mapped to the SARS-CoV-2 structure, where blue and red indicate positions where mutations increase or decrease serum binding, respectively.

bioRxiv preprint doi: https://doi.org/10.1101/2023.09.12.557371; this version posted September 13, 2023. The copyright holder for this preprint (which was not certified by peer review) is the author/funder, who has granted bioRxiv a license to display the preprint in perpetuity. It is made available under aCC-BY 4.0 International license.

Table S1 *Rhinolophus* ACE2 alleles and contact residues

			ACE2-RBD Contact Residues													
**Species**	**Isolate #**	**Genbank**	**24**	**27**	**30**	**31**	**34**	**35**	**38**	**41**	**42**	**45**	**83**	**330**	**353**	**355**
R. affinis	9479	MT394208.1	R	I	D	N	H	E	D	Y	Q	L	Y	N	K	D
R. affinis	787	MT394203.1	R	I	D	N	R	E	E	Y	Q	L	Y	N	K	D
R. sinicus	3364	MT394200.1	R	I	D	E	S	E	D	Y	K	L	Y	N	K	D
R. sinicus	WJ4	MT394181.1	L	I	D	E	F	E	N	Y	Q	L	Y	N	K	D
R. sinicus	-	GQ262791.1	L	I	D	E	S	E	N	Y	Q	L	Y	N	K	D
R. sinicus	1446	MT394194.1	R	T	D	E	S	E	N	Y	Q	L	Y	N	K	D
R. sinicus	WJ1	MT394187.1	R	I	D	T	S	E	D	Y	Q	L	Y	N	K	D
R. sinicus	1434	MT394197.1	R	M	D	T	S	E	D	Y	Q	L	Y	N	K	D
R. sinicus	3358	MT394193.1	E	M	D	K	T	K	D	H	Q	L	Y	N	K	D
R. sinicus	1438	MT394184.1	E	I	D	K	T	K	D	H	Q	L	Y	N	K	D
R. alcyone	-	KR559016.1	L	I	D	N	S	E	N	H	Q	L	F	N	K	D
R. landeri	-	KR559015.1	L	T	D	D	S	A	N	Y	Q	L	F	N	K	D
R. ferrumequinum	-	XM_033107295.1	L	K	D	F	S	E	N	H	Q	L	F	N	K	D
R. ferrumequinum	-	FJ598617.1	L	T	E	K	T	E	D	Y	Q	L	Y	K	K	D
Human	-	BAB40370.1	Q	T	D	K	H	E	D	Y	Q	L	Y	N	K	D

bioRxiv preprint doi: https://doi.org/10.1101/2023.09.12.557371; this version posted September 13, 2023. The copyright holder for this preprint (which was not certified by peer review) is the author/funder, who has granted bioRxiv a license to display the preprint in perpetuity. It is made available under aCC-BY 4.0 International license.

Table S2 CryoEM data collection and refinement statistics.

	PRD-0038 RBD - *R. alcyone* ACE2 EMD-41786	PRD-0038 RBD - *R. alcyone* ACE2 (Local refinement) PDB 8U0T EMD-41784	PRD-0038 S PDB 8U29 EMD-41842	PRD-0038 S (NTD local refinement) EMD-41843
**Data collection and processing**				
**Magnification**	105,000	105,000	105,000	105,000
**Voltage (kV)**	300	300	300	300
**Electron exposure (e^{-}/Å^2)**	60	60	60	60
**Defocus range (μm)**	-0.2 - -3.0	-0.2 - -3.0	0 - -3.0	0 - -3.0
**Pixel size (Å)**	0.843	0.843	0.843	0.843
**Symmetry imposed**	C2	C1	C3	C1
**Final particle images (no.)**	304,806	284,934	103,347	310,041
**Map resolution (Å)**	3.5	3.2	2.8	2.9
**FSC threshold**	0.143	0.143	0.143	0.143
**Map sharpening *B*factor (Å^2)**	-133	-118	-82.6	-89.7
**Validation**				
**MolProbity score**		1.08	1.28	
**Clashscore**		1.62	1.69	

bioRxiv preprint doi: https://doi.org/10.1101/2023.09.12.557371; this version posted September 13, 2023. The copyright holder for this preprint (which was not certified by peer review) is the author/funder, who has granted bioRxiv a license to display the preprint in perpetuity. It is made available under aCC-BY 4.0 International license.

**Poor rotamers (%)**		**0.47**	**1.13**	
**Ramachandran plot**				
**Favored (%)**		**97.02**	**95.44**	
**Allowed (%)**		**2.86**	**4.38**	
**Disallowed (%)**		**0.12**	**0.19**	

Table S3 Representative K_D values determined from BLI binding analysis.

	K_D (M)	K_D Error	k_a (1/Ms)	k_a Error	k_{dis} (1/s)	k_{dis} Error
**PRD-0038 WT RBD : monomeric *R. alcyone* ACE2**	5.07E-08	1.64E-10	1.64E+05	5.10E+02	8.33E-03	7.80E-06
**PRD-0038 T487W RBD : monomeric *R. alcyone* ACE2**	2.12E-08	3.96E-11	1.24E+05	2.18E+02	2.62E-03	1.63E-06
**PRD-0038 WT RBD : monomeric *R. landeri* ACE2**	N.D.	N.D.	N.D.	N.D.	N.D.	N.D.
**PRD-0038 T487W RBD : monomeric *R. landeri* ACE2**	1.88E-07	4.22E-10	4.49E+04	9.64E+01	8.43E-03	5.62E-06

* N.D. - not determined due to weak binding
